# A Novel Kelch-Like-1 Is Involved in Antioxidant Response by Regulating Antioxidant Enzyme System in *Penaeus vannamei*

**DOI:** 10.3390/genes11091077

**Published:** 2020-09-15

**Authors:** Xue-Li Qiao, Qing-Jian Liang, Yuan Liu, Wei-Na Wang

**Affiliations:** Guangzhou Key Laboratory of Subtropical Biodiversity and Biomonitoring, Guangdong Provincial Key Laboratory for Healthy and Safe Aquaculture, Key Laboratory of Ecology and Environmental Science in Guangdong Higher Education, College of Life Science, South China Normal University, Guangzhou 510631, China; qfxlong@163.com (X.-L.Q.); qingjian_liang@sina.cn (Q.-J.L.); wangwn@scnu.edu.cn (W.-N.W.)

**Keywords:** *Pv*Kelch-like-1, *Penaeus vannamei*, cadmium, antioxidant

## Abstract

Heavy metals are typical cumulative pollutants that can enter and poison the human body through the food chain. However, the molecular mechanism of heavy metal-induced oxidative stress is unclear. In this study, we characterize PvKelch-like-1 from *P. vannamei* and explore its antioxidant roles in immune regulation of crustaceans. *Pv*Kelch-like-1 full length contains 2107 nucleotides, consists of a 5′ untranslated region (UTR) of 79 bp, a 3′ UTR of 180 bp, and a ORF of 1848 encoded 615 amino acids, which contain a BTB, BACK and Kelch motif, putative molecular mass and isoelectric point were 69 KDa and 6.54. PvKelch-like-1 mRNA was ubiquitously expressed in all detected tissue of *P. vannamei*, and mRNA expression levels were significantly up-regulated from 6 to 24 h after cadmium stress and reached the highest level (3.2-fold) at 12 h in the hepatopancreas. Subcellular localization analysis revealed that PvKelch-like-1 was localized in the nucleus. Silencing PvKelch-like-1 significantly increased reactive oxygen species (ROS) (1.61-fold) production and DNA damage (1.32-fold) in the shrimp hemolymph, and significantly decreased total hemocyte counts (THC) (0.64-fold) at 6 h in hemolymph. Additionally, the antioxidant genes PvCAT (0.43-fold), PvMnSOD (0.72-fold), PvGST (0.31-fold) and PvGPx (0.59-fold) at 6 h were decreased significantly in PvKelch-like-1 silenced shrimp after cadmium stress. Overexpression of PvKelch-like-1 has the opposite results in enzyme activity. The SOD (2.44-fold) and CAT (2.19-fold) activities were significantly increased after overexpressing PvKelch-like-1. These results suggest that PvKelch-like-1 plays a vital role in shrimp innate immune defense by positively regulating the expression of antioxidant enzyme genes to respond to cadmium stress.

## 1. Introduction

In recent years, heavy metal pollution has become increasingly serious in China, and has become an important pollution factor for aquatic water environments, which is causing serious harm to farmed animals. Cadmium (Cd) may endanger the growth and development of aquatic organisms through toxic effects [1,2]. Accumulating evidence suggests that cadmium can damage the immune system of shrimp, trigger inhibition of physiology of shrimp and cause huge economic losses in the shrimp farming industry [3,4,5]. Recent studies on heavy metal pollutants have focused mainly on the effects of the acute toxicity and antioxidant system enzymes of organisms. However, there are few studies on the mechanism of detoxification of organisms.

Over the past thirty years, marine invertebrates subjected to various harmful stimuli induced oxidative stress have been increasingly studied. Recent studies have shown that reactive oxygen species (ROS) as a secondary messenger is crucial in the response to environmental stresses, growth and development [6,7]. However, excessive ROS can cause imbalance in the oxidation system and the antioxidant system, leading to oxidation damage [8]. Our initial findings are that *Pv*DJ-1 is of critical importance in the antioxidant function of shrimp [9]. Meanwhile, DJ-1 as transcription factors has been confirmed in the involvement of the Kelch-like ECH-associated protein 1 (Keap1)-Nrf_2_ antioxidant signaling pathway in mammals or fruit flies [10,11,12].

Keap1 belongs to the BBK protein family because it contains BTB, BACK and Kelch domains. The BTB domain is a conserved domain comprising approximately 115 hydrophobic amino acids, which play a functional role in mediating protein–protein interactions and is capable of protein self-association or interacting with other non-BTB proteins [13,14]. Kelch domains (Kelch-repeat motifs) consist of 3–7 Kelch, one of which contains 44–56 amino acids and it was found for the first time in Drosophila melanogaster [15]. Various proteins containing Kelch domain have been found in vertebrates, which belong to the Kelch-repeat superfamily protein [16]. The Kelch domain generally exists at the C-terminal of the protein, and conserved BTB or other domains exist at the N-terminal. At present, research on the BBK protein family focuses on the following aspects: cell morphology [17], protein ubiquitination degradation [18], and signal transduction [19,20]. It has been found in both vertebrates and fruit flies that the Keap1-Nrf2 signaling pathway plays an important role in the antioxidant function [21,22,23]. However, there is no report on this signaling pathway in shrimp.

Kelch-like ECH-associated protein 1 (Keap1) is an important model in the study of BBK family proteins. Therefore, this paper studied the antioxidative function of *Pv*Kelch-like-1 under cadmium stress, and combined with the mRNA regulation relationship between *Pv*DJ-1 and *Pv*Kelch-like-1, it has enriched the research on the antioxidant system of *P. vannamei* and provided a theoretical basis for solving the oxidative stress of shrimp in response to environmental stress.

## 2. Materials and Methods

### 2.1. Animal

*P. vannamei* (2–3 g in weight) were used in this study. The shrimp were maintained in 120 × 120 × 80 cm aquariums of seawater (5 ‰ salinity, pH 7.4 and 24 to 25 °C) and acclimatized for 3 days prior to the experiments.

### 2.2. CDNA Synthesis and RT-qPCR

Total RNA was extracted using Trizol (Invitrogen, Carlsbad, CA, USA) according to manufacturer’s instructions. RNA concentration was determined using NanoDrop 2000 (Montchanin, DE, USA), and RNA integrity was verified by 1% agarose gels. cDNA was synthesized with a PrimeScript ^TM^ RT-PCR Kit (TaKaRa, Shiga, Japan) following the manufacturer’s instructions. The mRNA expression level was determined by quantitative real-time PCR (qRT-PCR) using the ABI 7500 system. The PCR products were further verified by sequencing, all primers are shown in Table S1. Three independent qPCR experiments were performed. Each experiment was performed in triplicate.

### 2.3. Identification PvKelch-Like-1

Based on the data of the *P. vannamei* genome, we cloned the full-length of PvKelch-like-1 with rapid amplification with SMARTer RACE 5′/3′ Kit (TAKARA, Japan), following manufacturer’s instructions and used primer (Table S1). We used BLAST program of NCBI (http://www.ncbi.nlm.nih.gov/blast) to analyze the sequence. Multiple sequence alignment was performed using the Bio-Edit (version 7.0.9.0).

A phylogenic tree was constructed using the MEGA 7.0 program with neighbor-joining methods at 1000 bootstrap replication.

### 2.4. Polyclonal Antibody Preparation

The ORF of *Pv*Kelch-like-1 was amplified by PCR, digested by a combination of restriction enzymes EcoRV and HindIII, and then used to construct the recombinant vector and transformed into *Escherichia coli* (*E. coli*) BL21 (DE3) (Table S1). After induction for 6 h with 1.0% isopropyl β-D-thiogalactopy ranoside (IPTG), we used His-Bind Resin (Novagen) to purify recombinant protein, according to the manufacturer’s instructions. Recombinant protein concentration was determined by the BCA method, correctness was reanalyzed by SDS-PAGE. Healthy Kunming mice (qualified number: Guangdong verification word 2007A064) were intraperitoneally injected with 100 ug recombinant protein emulsified with Freund’s complete adjuvant (Sigma) initially. Then, they were injected three times with emulsified Freund’s incomplete adjuvant (Sigma) at intervals of one week. Antisera were harvested seven days after the last injection from ear veins and stored at −80 °C. Each experiment was performed in triplicate.

### 2.5. Tissue-Specific Expression and Immunofluorescence

The healthy shrimp’s hepatopancreas, heart, stomach, eyestalk, gill, intestine, hemolymph, foot and muscle were collected. The real-time PCR was performed to analyze the expression pattern. All primers are shown in Table S1.

The muscle tissue of the normal shrimp was prepared in 4% paraformaldehyde, and then the green fluorescent protein was labeled with the obtained antibody. After incubation, the nucleus was stained with DAPI, and the distribution of *Pv*Kelch-like-1 protein was observed under a fluorescence microscope. Each experiment was performed in triplicate.

### 2.6. Cadmium Challenge

Sixty healthy *P. vannamei* individuals were randomly separated into two groups. One group was the challenge with 4.25 µM/L CdCl_2_ (Kermel, Tianjin, China), the other group as controls. Each group was performed in triplicate. The hepatopancreases were collected at 0, 1.5, 3, 6, 12 and 24 h post challenge, and stored at −80 °C for RNA extraction.

### 2.7. Overexpression of PvKelch-Like-1 in S2 Cell

Drosophila S2 cells (Invitrogen, USA) were cultured in Schneider’s Drosophila media (Gibco, Gaithersburg, MD, USA) containing 10% fetal bovine serum (FBS, Gibco, USA) at 28 °C. The recombination plasmid pAc5.1-*Pv*Kelch-like-1-V5/HisB or pAc5.1-V5/HisB was mixed with FuGENE^®^ HD Transfection Reagent (Promega, San Luis Obispo, CA, USA), and then transfected in S2 cells according to the manufacturer’s protocol. After incubation for 48 h, cells were collected. The enzyme activity was detected according to the Total Superoxide Dismutase Assay Kit with WST-8 (Beyotime, Shanghai, China) and Catalase Assay Kit (Beyotime, China) according to the manufacturer’s protocol. Each experiment was performed in triplicate.

### 2.8. Silenced PvKelch-Like-1 In Vivo

Double RNA was obtained in vitro using the T7 RiboMAX ^TM^ Express RNA Production System (Promega, USA) according to the manufacturer’s protocol. Then, the double RNA concentration was detected with application of NanoDrop 2000 and confirmed by 1.2% agarose gel. Finally, the dsRNA were stored at −80 °C.

Sixty healthy shrimp were randomly separated into 2 groups. Then, equal amounts of double RNA was injected intramuscularly with 10 ug (1 μg/μL solution) (ds*Pv*Kelch-like-1 and dsGFP as a control). Each group was performed in triplicate. The hepatopancreases were collected 0, 1, 2, 3, and 4 d post injection, and stored at −80 °C for RNA extraction. RT-qPCR was performed to analyze the interference efficiency.

The hepatopancreases were collected 0, 1.5, 3 and 6 h after the cadmium challenge. Each experiment was performed in triplicate. Hepatopancreas was used to detect the expression of antioxidant enzyme genes.

### 2.9. Comet Assays and Total Hemocyte Counts (THC)

The hemolymph samples were mixed into low melting agarose after being diluted to 10^5^ cells mL^−1^, and were applied to the slides pre-plated with normal melting agarose, placed in the lysing buffer for 2 h at 4 °C, and then the slides experienced electrophoresis at 20 V and then 200 mA for 25 min of ice-cold, cold neutralization buffer twice for 10 min then dehydrated in ethanol for 15 min and stored in the dark. Then, we used SYRB Green I (1:10,000) staining, and the cells were observed using fluorescence microscopy (Leica, Frankfurt, Germany). The CASP image analysis software measures the comet tail moment (OTM).

The total hemocyte counts (THC) used a hemocytometer and a light microscope (Olympus, Beijing, China) to measure, according to the manufacturer’s protocol.

### 2.10. ROS Accumulation

The hemolymph ROS accumulation was detected with a DCFH-DA kit (Beyotime, China), according to the manufacturer’s instructions. Briefly, the hemolymph cells were incubated at 37 °C for 20 min with 10 μM DCFH-DA. After PBS elution twice, the cells were detected with a FACS AriaIII flow cytometer system (BD Biosciences, New York, NY, USA). Each experiment was performed in triplicate.

### 2.11. Western Blotting

The tissue was lysed in RIPA buffer (Beyotime, China), containing 1 mm PMSF for 30 min at 4 °C, and centrifuged at 12,000× *g* for 15 min at 4 °C. The tissue lysate was mixed with 5× SDS-PAGE buffer (Beyotime) and boiled for 10 min. Then, 30 μg of each sample was subjected to 10% SDS-PAGE and Western blotting, as previously reported [24]. The primary antibody diluted 1:1000 and secondary antibody diluted 1:2000 in 0.5% BSA-TBST. Following a further four 10 min washes in TBST, the membranes were detected with 5-bromo-4-chloro-3-indolyl-phosphate (BCIP)/nitroblue tetrazolium (NBT) substrate (Sangon Biotech, Shanghai, China). Each experiment was performed in triplicate.

### 2.12. Statistical Analysis

All data were presented as means ± SD. All data were performed using SPSS 20.0. Significant differences between means were analyzed with a one-way ANOVA analysis, *p* Values < 0.05 were considered statistically significant.

## 3. Results

### 3.1. Characterization of PvKelch-Like-1

The *Pv*Kelch-like-1 full length sequence consisted of 2107 nucleotides and contained an 1848 bp ORF that encoded a putative protein with 615 amino acids, a 5′ untranslated region (UTR) of 79 bp, and a 3′ UTR of 180 bp. The molecular mass and isoelectric point (pI) of this protein were predicted at 69 KDa and 6.54. It is predicted by the SMART program (http://smart.embl-heidelberg.de/, use default parameters) that the *Pv*Kelch-like-1 protein contains three domains: BTB at positions 76–173, BACK at positions 178–279, and Kelch at positions 320–606 (Figure S1A). The multiple sequence alignment revealed that the PvKelch-like-1 three domains BTB, BACK and Kelch are conservative. PvKelch-like-1 amino acid sequence is most similar to that of *Cryptotermes secundus* (accession no. XP_023726037.1) with 64.37% and *Zootermopsis nevadensis* (accession no. XP_021934020.1) with 64.02%, *Pv*Kelch-like-1 shares 36.08–64.37% similarity with other known sequences in this family (Figure S1B). Moreover, a phylogenic tree showed that *Pv*Kelch-like-1 has separated branches including vertebrates and invertebrates and was close to the arthropod (Figure S1C).

### 3.2. Preparation of Polyclonal Antibody

**Figure 1:**
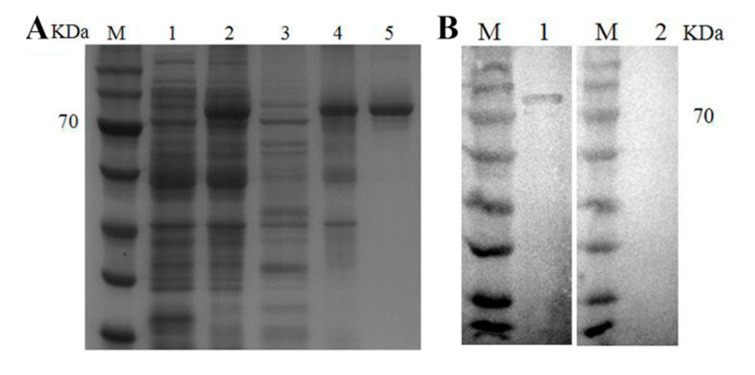
Preparation of polyclonal antibody. (**A**) Recombinant expression of *Pv*Kelch-like-1. Lane M, protein marker; Lane 1, control group with no isopropyl β-D-thiogalactopy ranoside (IPTG); Lane 2, control group inducted for 2 h with 1.0 mM IPTG; Lane 3, the sample of supernatant; Lane 4, dissolution of precipitation; Lane 5, purified protein; (**B**) Western blot analysis of the polyclonal antibody. Lane M, protein marker; Lane 1, *Pv*Kelch-like-1 recombinant protein incubated with experimental group serum; Lane 2, *Pv*Kelch-like-1 recombinant protein incubated with control group serum.

The recombinant *Pv*Kelch-like-1 protein has a significant band of approximately 88 KDa target protein (Figure 1A), and conformed to the expected results. Western blotting recognized a constituent of recombinant protein with an apparent molecular mass of 88 kDa, corresponding to that predicted from the *Pv*Kelch-like-1 (Figure 1B).

### 3.3. Subcellular Localization and Tissue-Specific Expression

**Figure 2:**
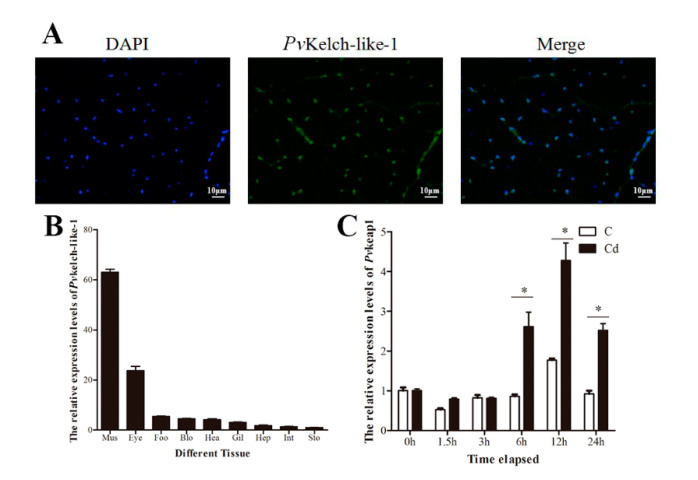
Subcellular localization and expression pattern of PvKelch-like-1. (**A**) Immunofluorescence analysis. Blue fluorescence is DAPI-stained nucleus of muscle cells, green fluorescence is the distribution of PvKelch-like-1 protein in cells. Pictures were taken at a magnification of 400 × fluorescence microscope; (**B**) the differential mRNA expression of PvKelch-like-1 in muscle (Mus), eyetalk (Eye), foot (Foo), hemolymph (Hem), hepatopancreas (Hep), gill (Gil), stomach (Sto), heart (Hea), intestine (Int). Vertical bars are presented as mean ± SD (*n* = 3); (**C**) expression of PvKelch-like-1 in the hepatopancreas of shrimp after cadmium challenge. Vertical bars represent the mean ± SD (*n* = 3). Different letters indicate statistically significant differences (*p* < 0.05) relative to normal shrimp (0 h). Asterisks indicate statistically significant differences (*p* < 0.05) between the two treatment groups.

The subcellular localization of *Pv*Kelch-like-1 was determined by a immunofluorescence assay. The results show that the *Pv*Kelch-like-1 localization nucleus of muscle cells use the polyclonal antibody of *P. vannamei* (Figure 2A). *Pv*Kelch-like-1 was detected in all tissue, with the highest expression in muscle (Figure 2B).

### 3.4. Expression Pattern of PvKelch-Like-1 after Cadmium Challenge

Expression of *Pv*Kelch-like-1 was analyzed in hepatopancreas samples after cadmium challenge of *P. vannamei*. The expression of *Pv*Kelch-like-1 rose gradually for the first 6 h, showing a 2.3-fold increase relative to the start of the experiment, at 12 h it reached maximum (3.2-fold) and then subsided slightly at 24 h after cadmium challenge (Figure 2C). In contrast, no significant change of *Pv*Kelch-like-1 expression was observed in the control shrimp.

### 3.5. PvKelch-Like-1 Participates Injury Response under Cadmium Stress

**Figure 3:**
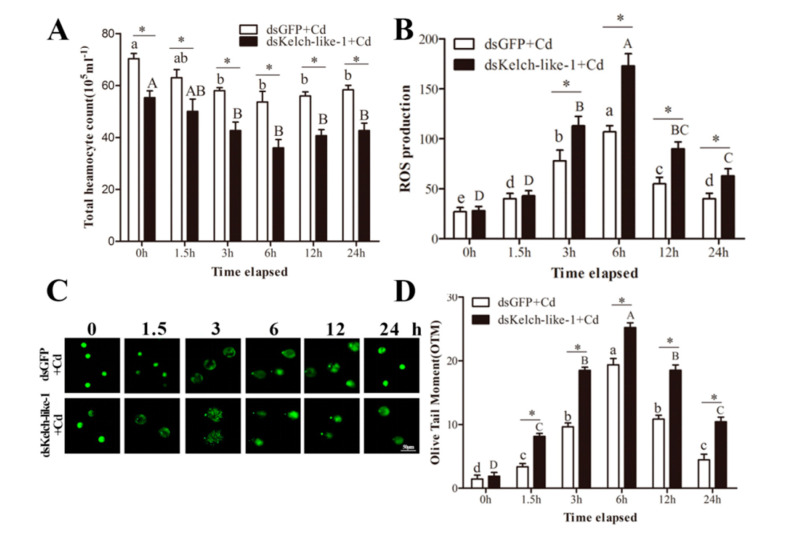
Effect of knockout *Pv*Kelch-like-1 in hemolymph. (**A**) Total hemocyte count; (**B**) endogenous reactive oxygen species (ROS) in the hemocytes; (**C**) images of DNA damage from the assayed hemocytes; (**D**) the change in DNA comet tail moment (OTM) value of hemocytes. Vertical bars represent the mean ± SD (*n* = 3). Different letters indicate statistically significant differences (*p* < 0.05) between Cd challenged and unchallenged shrimp (0 h). Asterisks indicate statistically significant differences (*p* < 0.05) between the two treatment groups.

The *Pv*Kelch-like-1 was knocked down (0.37-fold, 0.42-fold and 0.47-fold compared with *GFP*-RNAi) at 2, 3 and 4 d after *Pv*Kelch-like-1 group in *P. vannamei*, respectively (Figure S2A). In contrast, the dsGFP group had no significant effects on PvKelch-like-1 expression. As shown in Figure 3A, the THC in *Pv*Kelch-like-1-silenced shrimp was significantly lower than in the control group at 0 h post challenge. The THC of both groups significantly decreased after cadmium challenge and reached its minimum at 6 h. As shown in Figure 3B, ROS production increased at first and then decreased after cadmium challenge in both *Pv*Kelch-like-1 silenced and control groups, and reached the maximum at 6 h. During the 3 h to 24 h after cadmium challenge, the ROS levels in the *Pv*Kelch-like-1 silenced group were significantly higher than the control group. Moreover, this decrease was more prominent in the *Pv*Kelch-like-1 silenced group than in the control group. As shown in Figure 3C,D, the OTM value describing hemolymph DNA damage for both groups was significantly increased compared to 0 h under cadmium challenge.

### 3.6. PvKelch-Like-1 Responds to Cadmium Stress by Regulating Antioxidant Enzyme System

The expression of the antioxidant enzyme gene was detected on the second day of *Pv*Kelch-like-1 silenced shrimp, and the results showed that the expression of *Pv*CAT, *Pv*MnSOD, *Pv*GPx and *Pv*GST in the *Pv*Kelch-like-1 silenced group were significantly decreased compared to the dsGFP silenced group (Figure S2B).

**Figure 4:**
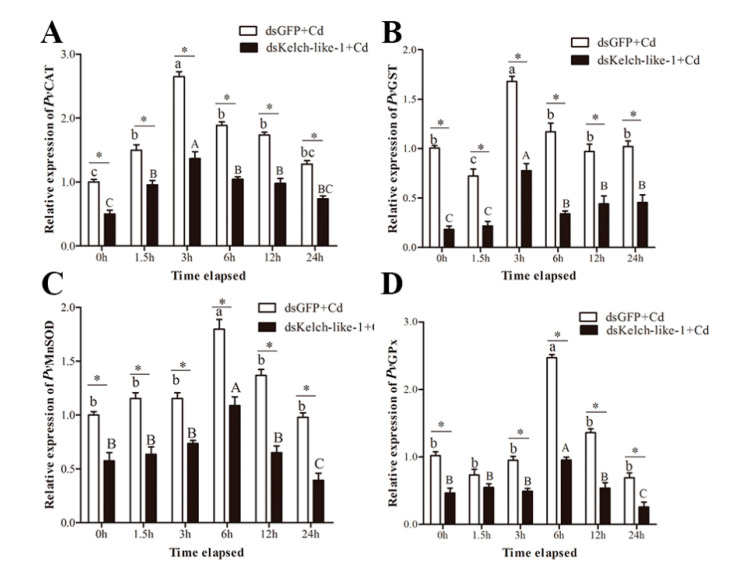
The mRNA expression of (**A**) *Pv*CAT (Catalase), (**B**) *Pv*MnSOD (Superoxide dismutase), (**C**) *Pv*GPx (Glutathione peroxidase) and (**D**) *Pv*GST (Glutathione S-transferase) in *Pv*Kelch-like-1-silenced shrimp after cadmium challenge. Vertical bars represent the mean ± SD (*n* = 3). Different letters indicate statistically significant differences (*p* < 0.05) between Cd challenged and unchallenged shrimp (0 h). Asterisks indicate statistically significant differences (*p* < 0.05) between the two treatment groups.

The expression of antioxidant enzymes in both groups showed significant differences under cadmium stress. The expression of *Pv*CAT and *Pv*GST reached the maximum at 3 h after cadmium challenge and then decreased, and the expression of *Pv*MnSOD and *Pv*GPx reached the maximum at 6 h after cadmium challenge and then decreased. Antioxidant enzymes gene *Pv*CAT, *Pv*MnSOD, *Pv*GPx and *Pv*GST expression were significantly decreased in the PvKelch-like-1 silenced group than in the control group at 0, 1.5, 3, 6, 12 and 24 h post challenge, respectively (Figure 4).

### 3.7. Effect of Enzyme Activity after Overexpression of PvKelch-Like-1 in S2 Cell

**Figure 5:**
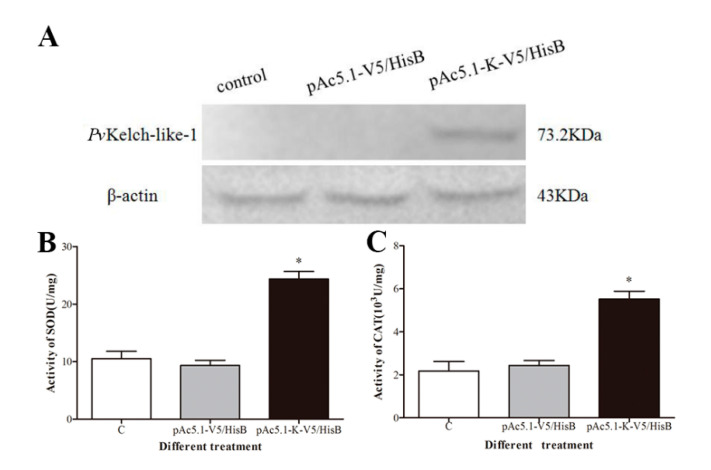
Effects of overexpression of *Pv*Kelch-like-1 on antioxidase activity. (**A**) Western blot detected *Pv*Kelch-like-1; (**B**) activity detection of SOD after overexpression of *Pv*Kelch-like-1; (**C**) activity detection of CAT after overexpression of *Pv*Kelch-like-1. Vertical bars represent the mean ± SD (*n* = 3). Asterisks indicate statistically significant differences (*p* < 0.05) between the three treatment groups.

*Pv*Kelch-like-1 recombinant eukaryotic protein with a molecular weight of 73.2 kDa was successfully expressed in S2 cells by Western blot (Figure 5A). The SOD and CAT activities were significantly increased compared to the transfected, with empty plasmids group after overexpressing PvKelch-like-1 in S2 cells (Figure 5B,C).

### 3.8. Relationship between PvKelch-Like-1 and PvDJ-1

**Figure 6:**
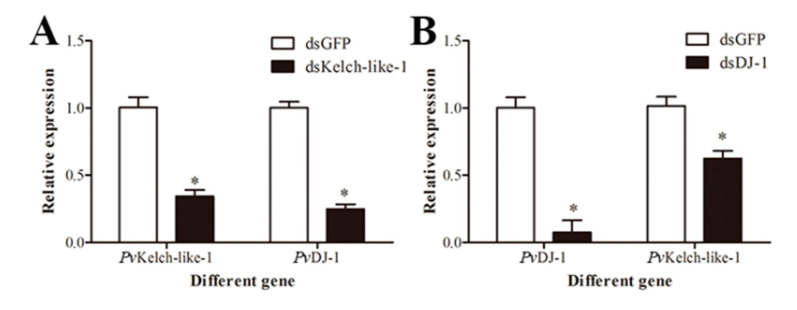
Regulatory relationship of *Pv*Kelch-like-1 and *Pv*DJ-1. (**A**) Expression of *Pv*DJ-1 in *Pv*Kelch-like-1-silenced shrimp; (**B**) expression of *Pv*Kelch-like-1 in *Pv*DJ-1-silenced shrimp. Vertical bars represent the mean ± SD (*n* = 3). Asterisks indicate statistically significant differences (*p* < 0.05) between the two treatment groups.

In order to verify whether there is a regulatory relationship between *Pv*Kelch-like-1 and *Pv*DJ-1, the expression of *Pv*DJ-1 was detected in *Pv*Kelch-like-1-silenced shrimp, and the expression of *Pv*Kelch-like-1 was detected in *Pv*DJ-1-silenced shrimp. The results show that *Pv*DJ-1 significantly decreased after silenced *Pv*Kelch-like-1 (Figure 6A), and the expression of *Pv*Kelch-like-1 significantly decreased after silenced *Pv*DJ-1(Figure 6B).

## 4. Discussion

The dynamic balance of oxidative stress is essential for the physiological activities of living organisms [25]. Studies have shown that when the external environmental factors change, the oxidative stress reaction of shrimp causes excessive ROS to damage the innate immune system of shrimp, including the antioxidant enzyme system [26,27,28]. At present, there is little basic research on the antioxidation system of shrimp. *Pv*DJ-1, in the previous work, has confirmed that it has significant antioxidant effects similar to vertebrates [9,29,30]. In this paper, cloning and functional studies reveal that *Pv*Kelch-like-1 in the *Pv*DJ-1 transcriptome occupies a position in the shrimp antioxidant system.

Sequence analysis revealed that *Pv*Kelch-like-1 belongs to the BBK family with poor nucleotide conservation. It is speculated that there are functional differences between aquatic organism BBK family genes and vertebrates. Tissue distribution analyses in *P. vannamei* showed that *Pv*Kelch-like-1 had the highest expression in the muscle, which was supposed to be related to its function in the Kelch domain. Kelch was found in Drosophila interacted with actin and had an important role in the growth and development [31,32].

Keap1, a cytoplasmic protein, is one of the most widely studied antioxidant-related genes in the BBK family [33,34] and itinteractes with the nuclear transcription factor Nrf_2_ to maintain redox homeostasis [35,36]. Immunofluorescence verified that the *Pv*Kelch-like-1 protein was localized in the nucleus of the shrimp cells, which was different from the cytoplasmic localization result of Keap1. BBK family proteins play an important role by localization in a different location [37,38]. KLHL31 protein is localized in both the nucleus and the cytoplasm to inhibit the transcriptional activities of TRE and SRE [39]. KLHL7 protein is expressed in the nuclei of neurons, and its antibodies can be used as paraneoplastic markers [40]. According to the localization results of *Pv*Kelch-like-1 protein, it is speculated that *Pv*Kelch-like-1 may be involved in nuclear transcriptional regulation.

It has been reported that cadmium exposure reduces the THC, induces DNA damage and ROS production of hemolymph in shrimp [41,42,43]. Our results found that the THC was significantly decreased, the DNA damage was significantly increased, and the ROS level was significantly increased in the *Pv*Kelch-like-1 silenced group, indicating that the ability to regulate oxidative stress of the hemolymph in shrimp under cadmium stress was impaired after *Pv*Kelch-like-1 was silenced. The hepatopancreas plays important roles in several metabolic processes and the immunoregulatory process in shrimp [44,45]. It was found that the hepatopancreas of shrimp developed lesions under heavy metal stress, and expression of antioxidant enzymes was induced [46,47,48]. Whether PvKelch-like-1 affect the expression of antioxidant enzyme gene in *P. vannamei.* In this study, expression of antioxidant enzymes gene significantly decreased in the *Pv*Kelch-like-1 silenced group compared with GFP silenced group after cadmium stress. Knockout Keap1 revealed a significant increase in the antioxidant enzyme gene HO-1 and Nrf_2_ [49,50]. Studies have shown that overexpression of the antioxidant-related gene Keap1 in vertebrate cells significantly reduces the expression of antioxidant enzymes, which is different from our results [51,52]. Our results found that the activity of antioxidant enzymes SOD and CAT was significantly increased after overexpressed *Pv*Kelch-like-1 in Drosophila S2 cells. All results reveal that *Pv*Kelch-like-1 positively regulates the expression of antioxidant enzyme genes under response to cadmium stress.

Recently published data suggest that DJ-1 is necessary for the Nrf2/Keap1 axis and antioxidant stress [10]. Preliminary investigation on the regulatory relationship between the antioxidant genes PvKelch-like-1 and PvDJ-1 was performed by detecting mRNA expression. The results show that there is a bidirectional regulation relationship between PvKelch-like-1 and PvDJ-1, but the specific regulatory mechanism needs further research.

In conclusion, we report the molecular characterization and functional investigation of *Pv*Kelch-like-1. Overexpression and silencing indicate that *Pv*Kelch-like-1 has a positive regulatory effect on the expression of antioxidant enzyme gene. Furthermore, the mRNA regulation relationship between *Pv*Kelch-like-1 and *Pv*DJ-1 is confirmed. These results reveal the function of the BBK family gene *Pv*Kelch-like-1 and provide a basis for the antioxidant system of *P. vannamei.*

## Supplementary Materials

The following are available online at https://www.mdpi.com/2073-4425/11/9/1077/s1, Figure S1 Characterization of *Pv*Kelch-like-1. (A) Nucleotide and deduced amino acid sequences of *Pv*Kelch-like-1. The translation initiation (ATG) and stop codons (TGA) are boxed. Three conserved sequences of *Pv*Kelch-like-1 protein domains are shaded in different gray (76-173 BTB; 178-279 BACK; 320-606 Kelch); (B) multiple sequence alignment of Kelch-like-1 protein between *P. vannamei* and other species. *Mus musculus* (BAA34639.1), *Drosophila melanogaster* (ABD14408.1), *Homo sapiens* (NP_987096.1), *Danio rerio* (NP_878284.2), *Latimeria chalumnae* (XP_005994374.1), *Limulus Polyphemus* (XP_013773778.1), *Wasmannia auropunctata* (XP_011697788.1). Similar amino acid residues (>50%) are labeled in gray while uniform amino acid residues are labeled in black; (C) phylogenetic tree of the *Pv*Kelch-like-1 was constructed with NJ method in MEGA 5.1 and a bootstrap analysis was performed using 1000 replicates to test the relative support for particular clades; Figure S2 Effect of silencing *Pv*Kelch-like-1 on antioxidant gene expression. (A) *Pv*Kelch-like-1 silenced efficiency detection in vivo; (B) the mRNA expression of *Pv*CAT, *Pv*MnSOD, *Pv*GPx and *Pv*GST in the hepatopancreas after *Pv*Kelch-like-1 knockdown.
