# Supplementary material for: A Novel Kelch-Like-1 Is Involved in Antioxidant Response by Regulating Antioxidant Enzyme System in Penaeus vannamei
Source: Genes (Basel). 2020 Sep 15;11(9):1077. doi: 10.3390/genes11091077 (PMC7564309; doi:10.3390/genes11091077)
Supplement: Supplementary file 1 [file genes-11-01077-s001.zip › genes-865674-suppls/Fig. S.docx]

Fig.S1


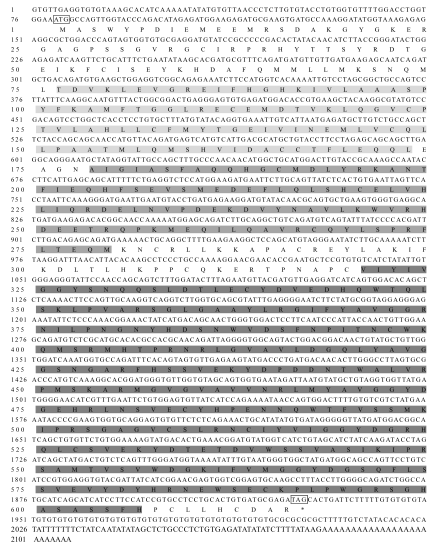


**(A)**


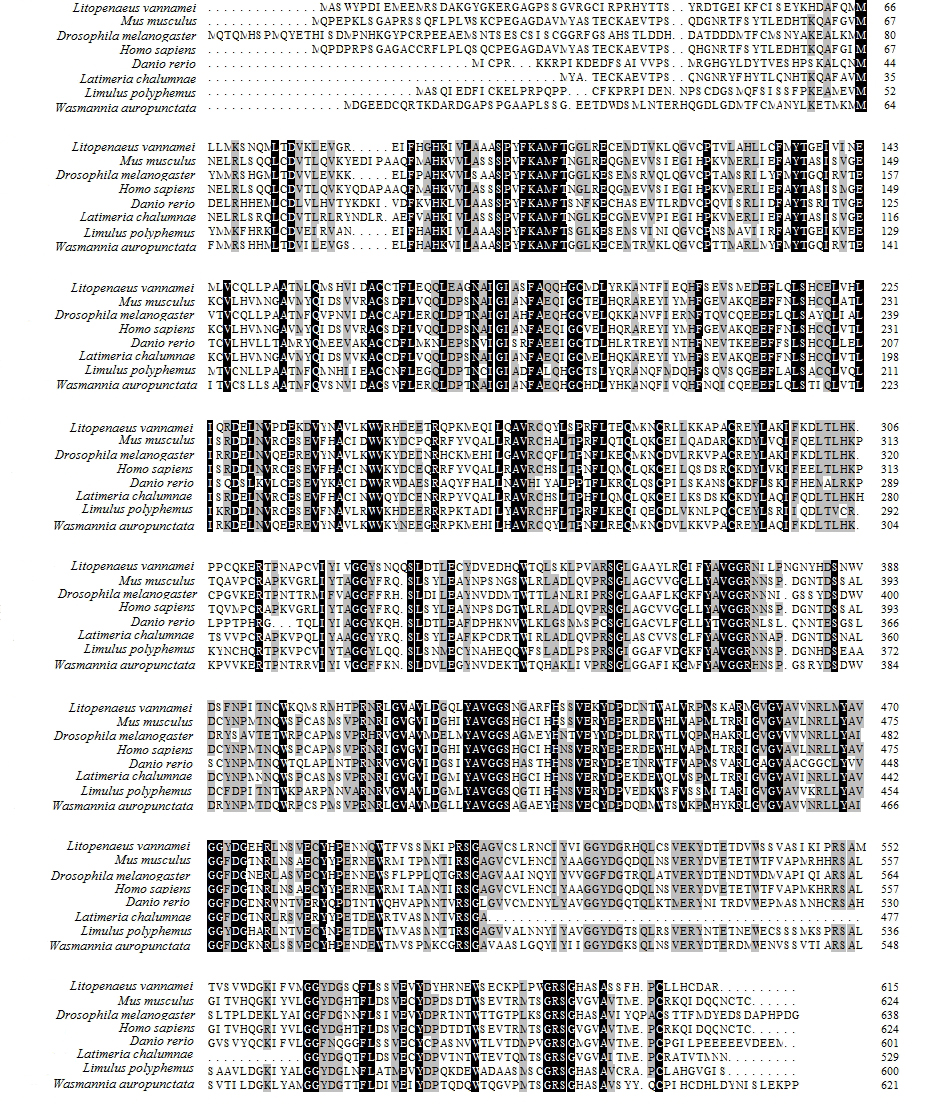


**(B)**


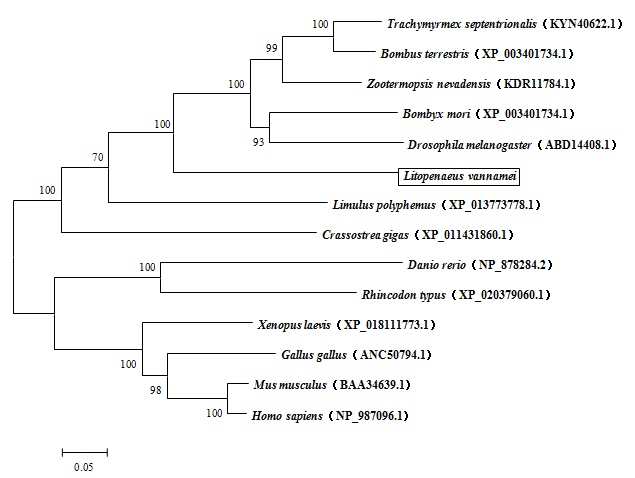


**(C)**

**Figure S1.** Characterization of *Pv*Kelch-like-1.(A) Nucleotide and deduced amino acid sequences of *Pv*Kelch-like-1. The translation initiation (ATG) and stop codons (TGA) are boxed. Three conserved sequences of *Pv*Kelch-like-1 protein domains are shaded in different gray (76-173 BTB; 178-279 BACK; 320-606 Kelch). (B) Multiple sequence alignment of Kelch-like-1 protein between *P. vannamei* and other species. *Mus musculus* (BAA34639.1), *Drosophila melanogaster* (ABD14408.1), *Homo sapiens* (NP_987096.1), *Danio rerio* (NP_878284.2), *Latimeria chalumnae* (XP_005994374.1), *Limulus Polyphemus* (XP_013773778.1), *Wasmannia auropunctata* (XP_011697788.1). Similar amino acid residues (> 50%) are labeled in gray while uniform amino acid residues are labeled in black. (C) Phylogenetic tree of the *Pv*Kelch-like-1 was constructed with NJ method in MEGA 5.1 and a bootstrap analysis was performed using 1000 replicates to test the relative support for particular clades.

Fig.S2


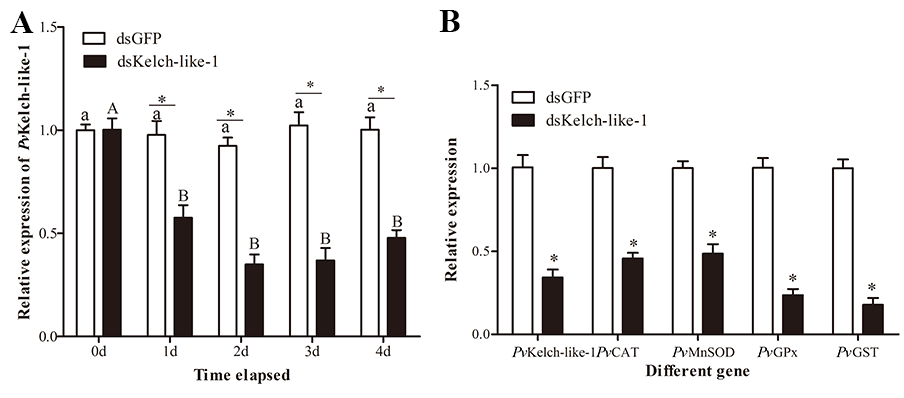


**Figure S2.** Effect of silencing *Pv*Kelch-like-1 on Antioxidant Gene Expression. **(A)** *Pv*Kelch-like-1 silenced efficiency detection *in vivo*; **(B)** The mRNA expression of *Pv*CAT, *Pv*MnSOD, *Pv*GPx and *Pv*GST in the hepatopancreas after *Pv*Kelch-like-1 knockdown.
