# Supplementary material for: A Novel Kelch-Like-1 Is Involved in Antioxidant Response by Regulating Antioxidant Enzyme System in Penaeus vannamei
Source: Genes (Basel). 2020 Sep 15;11(9):1077. doi: 10.3390/genes11091077 (PMC7564309; doi:10.3390/genes11091077)
Supplement: Supplementary file 1 [file genes-11-01077-s001.zip › genes-865674-suppls/Table S1.docx]

Table S1

**Table S1.** Sequences of PCR primers.

| **Primer** | **Sequence (5′-3′)** |
| --- | --- |
| **For cDNA cloning** |  |
| *Pv*Kelch-like-1-F | ATGGCCATGGTACCCAGGT |
| *Pv*Kelch-like-1-R | CTGCACTGTGATGCGAGATAG |
| UPM long | CTAATACGACTCACTATAGGGCAAGCAGTGGTATCAACGCAGAGT |
| UPM short | AAGCAGTGGTATCAACGCAGAGT |
| *Pv*Kelch-like-1-5′RACE-GSP1 | CCTCACCCACTTCAGCACTGCGTTGT |
| *Pv*Kelch-like-1-5′RACE-GSP2 | AGGAGGTGAGCCAGGACTGTCGGACAT |
| *Pv*Kelch-like-1-3′RACE-GSP1 | CACGCAACAGATTAGGGGTGGCAGT |
| *Pv*Kelch-like-1-3′RACE-GSP2 | GGTCGGAGTGCAAGCCTTTACCTTGG |
| **For Prokaryotic expression** |  |
| pET-*Pv*Kelch-like-1-F | GCGCGCGCGATATCATGGCCAGTTGGTACCCA |
| pET-*Pv*Kelch-like-1-R | GCGCGCGCAAGCTTTTAATGATGATGATGATGATGTCTCGCATCACAGTGCAG |
| **For overexpression** |  |
| pAc5.1- *Pv*Kelch-like-1-F | TGGTGGAATTCTGCAGATATCATCAAAATGGCCAGTTGGTACCCA |
| pAc5.1- *Pv*Kelch-like-1-R | CGCGGGCCCTCTAGACTCGAGGCTCTCGCATCACAGTGCAG |
| **For RNAi** |  |
| ds*Pv*Kelch-like-1-F-T7 | TAATACGACTCACTATAGGGCAGTGCTGAAGTGGGTGA |
| ds*Pv*Kelch-like-1-F | GCAGTGCTGAAGTGGGTGA |
| ds*Pv*Kelch-like-1-R-T7 | TAATACGACTCACTATAGGATCTGTTGCGTGGCGTGT |
| ds*Pv*Kelch-like-1-R | ATCTGTTGCGTGGCGTGT |
| dsGFP-F-T7 | TAATACGACTCACTATAGGGTGCCCATCCTGGTCGAGCT |
| dsGFP-F | GTGCCCATCCTGGTCGAGCT |
| dsGFP-R-T7 | TAATACGACTCACTATAGGTGCACGCTGCCGTCCTCGAT |
| dsGFP-R | TGCACGCTGCCGTCCTCGAT |
| **For RT-PCR** |  |
| *Pv*CAT-F | GAGGCCGTCTACTGCAAGTT |
| *Pv*CAT-R | GAGGGGTAATCGCCACTTGA |
| *Pv*MnSOD-F | GCGTTGGAGTGAAAGGCTCT |
| *Pv*MnSOD-R | TCACGTAATCTGCACGGAGG |
| *Pv*GPx-F | AGGGACTTCCACCAGATG |
| *Pv*GPx-R | CAACAACTCCCCTTCGGTA |
| *Pv*GST-F | AAGATAACGCAGAGCAAGG |
| *Pv*GST-R | TCGTAGGTGACGGTAAAGA |
| *Pv*β-actin-F | AGATGACCACCGCTGCTTC |
| *Pv*β-actin-R | ATGTCCACGTCGCACTTCAT |
